# Plasma biomarkers of neuroinflammation and neurodegeneration in relation to cerebral glucose hypometabolism in autosomal‐dominant Alzheimer's disease

**DOI:** 10.1002/alz70856_103647

**Published:** 2025-12-24

**Authors:** Catarina Tristão‐Pereira, David Fernando Aguillón Niño, Ana Y Baena, Natalia Londono, Lusiana Martinez, Sergio Alvarez, Monica Vidal, Vincent Malotaux, Bing He, Averi Giudicessi, Randy Medrano, Nikole A Bonillas Félix, Clara Vila‐Castelar, Edmarie Guzman‐Velez, Steven E Arnold, Bernard J Hanseeuw, Eric M. Reiman, Yakeel T. Quiroz

**Affiliations:** ^1^ Massachusetts General Hospital, Harvard Medical School, Boston, MA, USA; ^2^ Department of Psychological & Brain Sciences, Boston University, Boston, MA, USA; ^3^ Grupo de Neurociencias de Antioquia, Universidad de Antioquia, Medellín, Colombia; ^4^ Grupo de Neurociencias de Antioquia, Facultad de Medicina, Universidad de Antioquia, Medellín, Colombia; ^5^ Grupo de Neurociencias de Antioquia, Facultad de Medicina, Universidad de Antioquia, Medellín, Antioquia, Colombia; ^6^ Hospital Pablo Tobón Uribe, Medellín, Antioquia, Colombia; ^7^ Hospital Pablo Tobón Uribe, Medellin, Colombia; ^8^ Boston University, Boston, MA, USA; ^9^ Institute of Neuroscience, Université Catholique de Louvain, Brussels, Belgium; ^10^ Banner Alzheimer's Institute, Phoenix, AZ, USA

## Abstract

**Background:**

Cerebral glucose hypometabolism, measured with ^18^F‐fluorodeoxyglucose (FDG) PET, is a hallmark of Alzheimer's disease (AD). While FDG‐PET changes are highly effective at differentiating dementia types, the pathological bases for metabolic changes are complex. Hypometabolism is often assumed to reflect decreased neuronal activity, but glucose uptake is not limited to neurons. Astrocytes consume about half of the glucose‐derived brain energy and have been recognized as major contributors to the FDG‐PET signal. This study investigates the differential contribution of plasma glial fibrillary acidic protein (GFAP), a protein upregulated in reactive astroglia, and neurofilament light chain (NfL), a neuron‐specific cytoskeleton component, to FDG‐PET in autosomal‐dominant AD.

**Method:**

We included 40 Presenilin‐1 E280A mutation carriers (37.9±7.6 years, 26 females, 5 cognitively impaired) and 37 cognitively unimpaired non‐carriers (39.5±6.9 years, 23 females) from the Colombia‐Boston (COLBOS) Biomarkers Study (Table 1). Plasma GFAP and NfL were quantified using Neurology 4‐Plex E Advantage Kits (Quanterix). FDG uptake was processed across Freesurfer‐derived regions of interest. Spearman correlation was used to examine the associations between plasma biomarkers and regional FDG uptake, correcting for multiple comparisons. Lasso regression and mediation analysis identified the plasma biomarker most strongly linked to global FDG‐PET uptake (Landau meta‐ROI).

**Result:**

Mutation carriers had higher plasma GFAP and NfL levels than non‐carriers (ps<0.001). Among carriers, both plasma GFAP and NfL showed negative correlations with FDG uptake, following a similar regional pattern that included temporo‐parietal regions (uncorrected). Notably, the associations with FDG uptake in the hippocampus remained significant after multiple comparisons correction (GFAP: p_adj_=0.014; NfL: p_adj_=0.005). In the joint Lasso model, plasma GFAP (b=‐0.082 [‐0.156,‐0.002]), but not plasma NfL (b=‐0.063 [‐0.137,0.032]), remained associated with global FDG uptake. Plasma GFAP showed a direct effect on global FDG uptake, independently of plasma NfL (indirect effect: *p* = 0.153; direct effect: *p* = 0.021).

**Conclusion:**

Our data suggest that the FDG‐PET signal may reflect both reactive astrogliosis and neuronal injury in AD, with the effect of reactive astrogliosis on hypometabolism being independent of neurodegeneration. With astrocytes interfacing neurovascular communication, underlying vascular or neuroinflammatory mechanisms could contribute to hypometabolism and early disease onset, calling for a more cautious interpretation of brain FDG‐PET imaging.

